# Treatment and fertility outcomes of moss-like endometriosis with hemorrhagic ascites: A case report

**DOI:** 10.1097/MD.0000000000041872

**Published:** 2025-03-21

**Authors:** Feng Xu, Yimeng Gao, Fengxi He, Miao Zhao, Aihua Li

**Affiliations:** a School of Clinical Medicine, Shandong Second Medical University, Weifang, China; b Department of Gynecology and Obstetrics, Liaocheng People’s Hospital, Liaocheng, China.

**Keywords:** fertility, hemorrhagic ascites, moss-like endometriosis, treatment

## Abstract

**Rationale:**

Endometrial tissue in the greater omentum with a large amount of hemorrhagic ascites is relatively uncommon. Endometriosis is similar to ovarian malignancy in cases of hemorrhagic ascites. Hysterectomy and bilateral oophorectomy are the only effective and clear treatments, and their indications are limited by patient age and fertility. Conservative medical treatment is a viable option.

**Patient concerns:**

A 28-year-old female came to our hospital for treatment because of the discovery of a pelvic mass for 10 months. After admission, the patient underwent surgical treatment and was discharged within 1 week. She received medical treatment for 3 years after discharge. After medication withdrawal, the patient became pregnant and gave birth to a child without recurrence during the follow-up.

**Diagnoses:**

Initial diagnosis on first admission were “‘Pelvic inflammatory mass?’ and ‘endometriosis?’.” After 10 months, the patient was readmitted to the hospital because of gradual enlargement of the mass, and was diagnosed with endometriosis.

**Interventions:**

The patient was treated with anti-infective rehydration therapy for the first time, and the patient was surgically treated for the second time, followed by gonadotropin-releasing hormone agonist (GnRH-α) and dienogest (DNG) treatment after surgery.

**Outcomes:**

After 6 cycles of GnRH-α treatment and 3 years of DNG treatment, a son was successfully born after discontinuation of the medication, and so far, there has been no recurrence or adverse reactions during the follow-up period.

**Lessons:**

Ascites is a rare manifestation of endometriosis and its diagnosis is difficult. Laparoscopy or exploratory laparotomy is required to confirm this diagnosis. Cyclic dysmenorrhea and abnormal menstruation warrant vigilance and should be investigated carefully. Hysterectomy and bilateral oophorectomy should be avoided as much as possible in patients with fertility needs and age adaptation, and symptoms can be successfully resolved with medical therapy.

## 1. Introduction

Hemorrhagic ascites is a rare complication of moss-like endometriosis. The first description of endometriosis-related ascites was reported by Brews in 1954.^[1]^ Charles first documented a case of endometriosis complicated with blood-stained ascites in 1957. To date, fewer than 100 reports of hemorrhagic ascites associated with endometriosis have been published. The diagnosis of moss-like endometriosis complicated by hemorrhagic ascites is relatively difficult and can result in different symptoms. It may present with similar disease processes, such as malignancy, pelvic infection, cirrhosis or trauma, tuberculous peritonitis, and Demons-Meigs syndrome, which can be easily misdiagnosed.^[1,2]^ Abdominal distension and pain are the main symptoms, along with other nonspecific symptoms, such as abdominal mass, nausea or vomiting, decreased appetite, weight loss, fatigue and discomfort, and hypovolemic shock.^[3]^ In severe cases, cachexia and encapsulated peritonitis have been reported, and some patients lack the typical symptoms or pelvic manifestations. After repeated diagnosis and treatment in different hospitals and even acceptance of anti-tuberculosis treatment, the patient was finally diagnosed by laparoscopic exploration and biopsy.^[4]^ CA125 is not specific to endometriosis and is not closely associated with ascites. Although the majority of patients show CA-125 levels > 35 U/mL, CA-125 levels can also be elevated in other diseases, such as ovarian cancer and pelvic inflammatory diseases. Interstitial cells within the peritoneum secrete CA-125, and CA-125 is released owing to interstitial cell hyperplasia. Hypertrophy is associated with endometriosis, and serum CA-125 levels were elevated in this case.^[5]^ Some articles reported the level of CA-125, in which 6 patients had normal levels and 14 patients had elevated biomarker CA-125 levels (>35 U/mL) ranging from 49 U/mL to >5000 U/mL. One patient initially had normal CA-125 levels, but their levels began to increase (455 U/mL) after ascites recurrence.^[2]^ However, a study by Magalhães et al found that the CA-125 level was increased in 2/3 of the patients, and MRI suggested a T1 high signal in the lesion area, all of which needed to be diagnosed by surgical exploration.^[6–8]^ More than half of the patients show endometriosis-related triad, detailed consultation is quite significant, and endometriosis should be 1 of the differential diagnoses of massive hemorrhagic ascites in women of reproductive age.^[9]^ A meta-analysis by Magalhães et al^[6]^ found that the median age of these patients was 29 years, and most of them were Black or Asian and mostly nullipara. The volume of ascites is usually large, with an average of 3 to 4 L, and is chocolate colored, sero-like, or bloody, and is also described as “brown green” and “limited” limited’. Most cases occur slowly, with a small number of cases being acute, and approximately 30% of cases with right pleural effusion.

## 2. Case report

**Figure 1.**
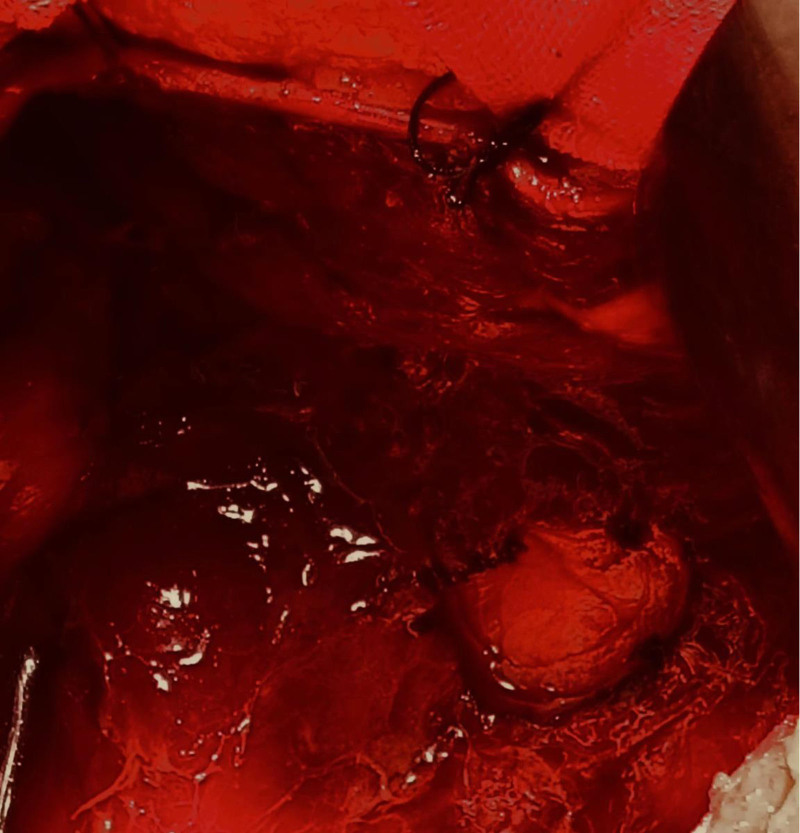
Hemorrhagic ascites.

**Figure 2.**
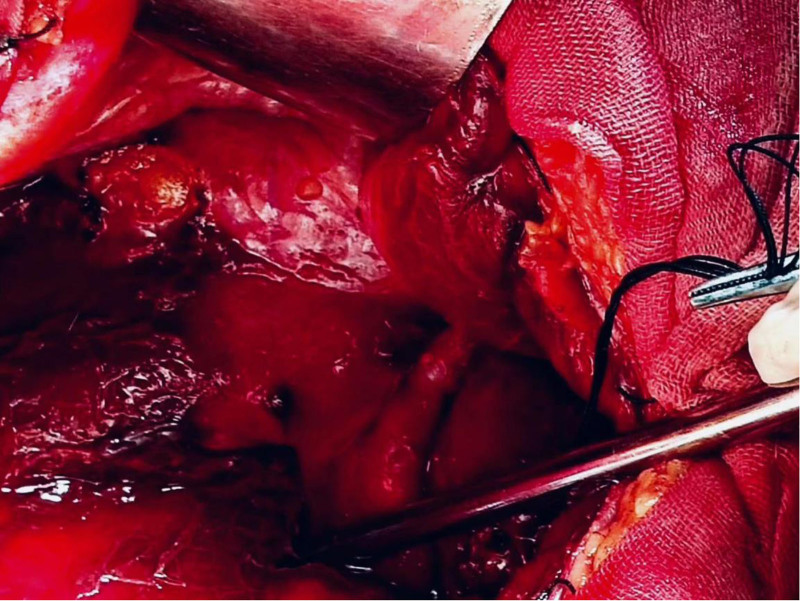
Hemorrhagic ascites.

**Figure 3.**
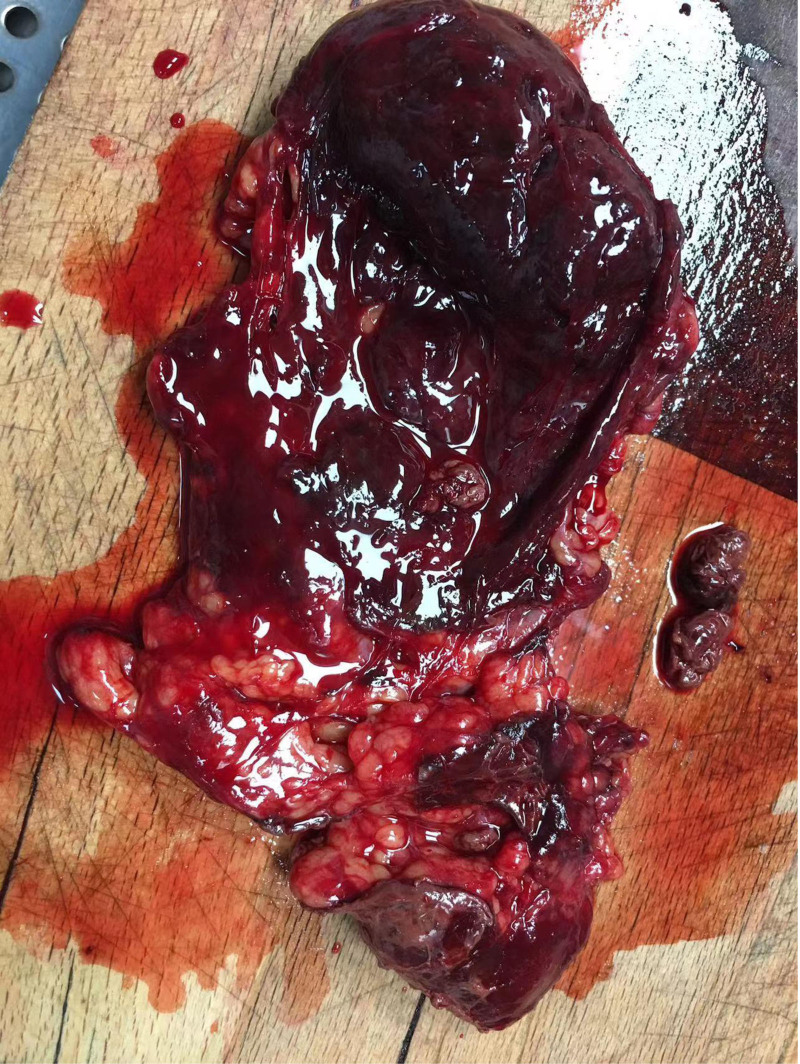
A gray-red mass with a diameter of approximately 8 cm is observed on the surface of the greater omentum, resembling rotten meat.

**Figure 4.**
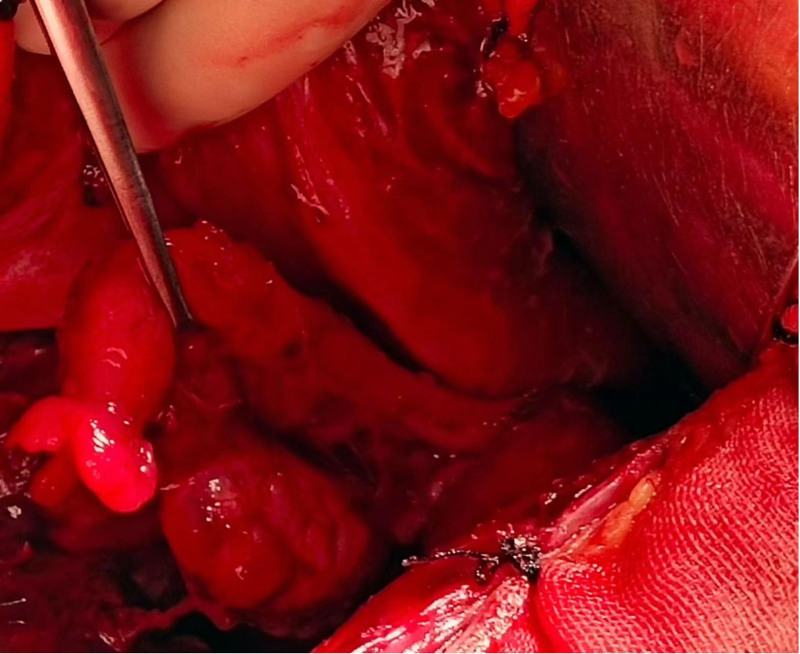
Diffuse “moss like” brown cellulose deposits were found on the surface of the uterus, bilateral adnexa, abdominal wall, intestinal tract, lateral groove of the rectum, and anterior wall of the rectum.

**Figure 5.**
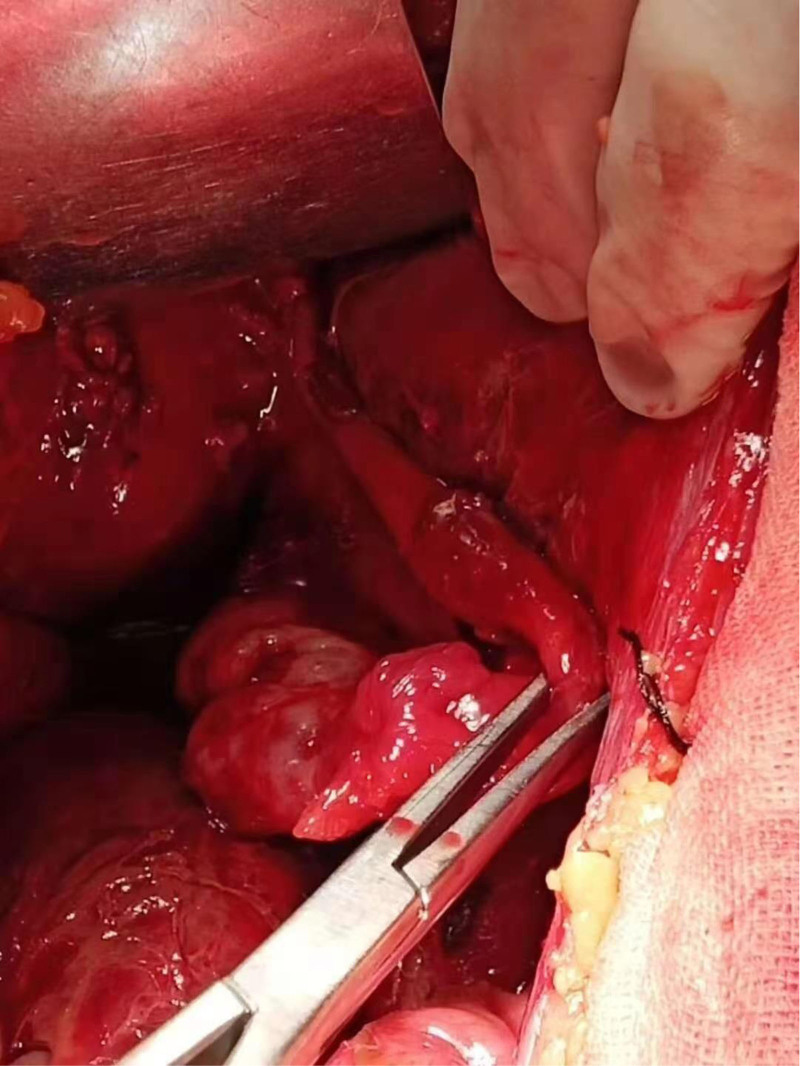
Diffuse “moss like” brown cellulose deposits were found on the surface of the uterus, bilateral adnexa, abdominal wall, intestinal tract, lateral groove of the rectum, and anterior wall of the rectum.

**Figure 6.**
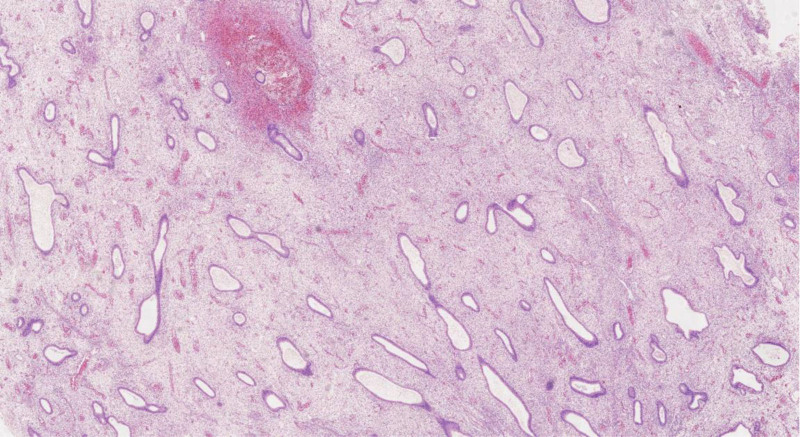
Postoperative pathologic investigation (HE × 40).

**Figure 7.**
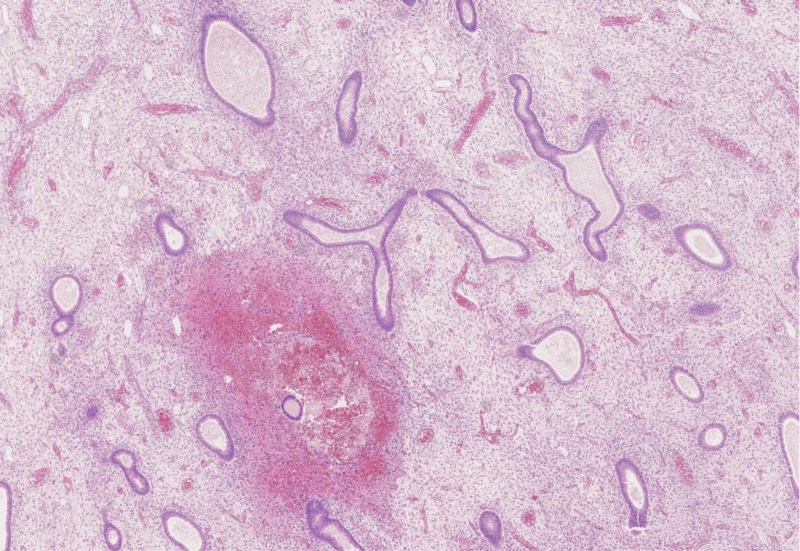
Postoperative pathologic investigation (HE × 100).

**Figure 8.**
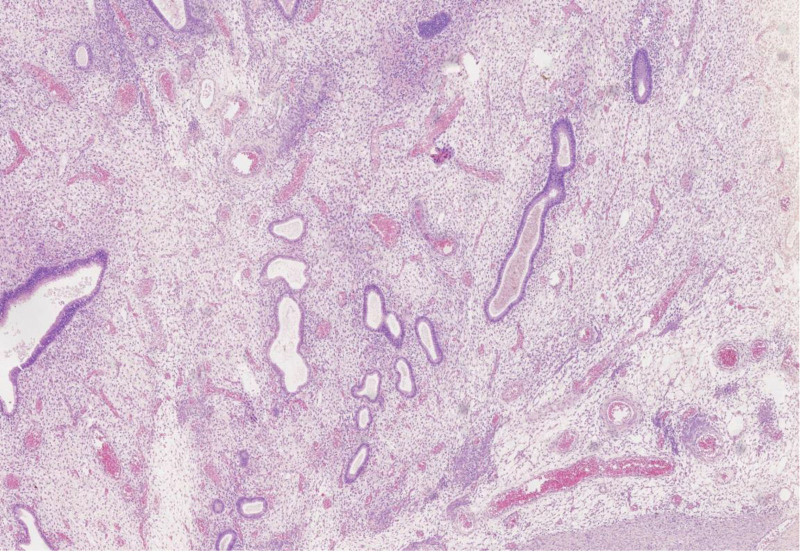
Postoperative pathologic investigation (HE × 100).

**Figure 9.**
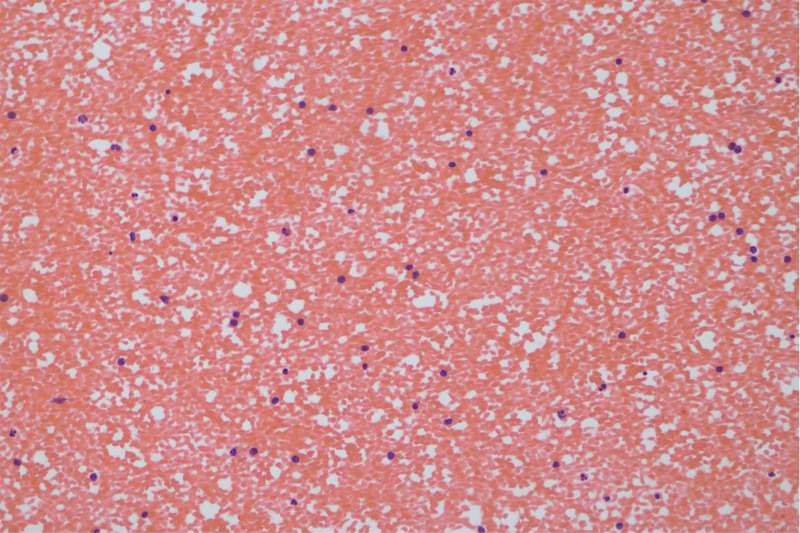
Cytology pathology of ascites (HE × 200).

A 28-year-old Asian woman was admitted to the hospital for the treatment of “a pelvic mass for 10 months” on November 11, 2018. The patient usually had regular menstruation, moderate menstrual volume, dysmenorrhea, and a G1P0L0A1. Gynecological examination: The rear of the uterus was approximately 8 cm in diameter with poor activity and tenderness, but no other abnormalities were noted. The patient had no obvious cause of abdominal pain during the first 10 months prior to admission. Ultrasound examination revealed a heterogeneous mass on the upper right side of the uterus with a diameter of approximately 4 cm, nodule of the greater omentum with a diameter of approximately 2 cm, and serum CA125 level of 416 mU/mL. Is it diagnosed as “endometriosis? Inflammatory Pelvic Masses”. Anti-infective rehydration therapy was administered and the patient was discharged with pain relief. Two weeks after discharge, the patient’s CA125 level decreased to 106 mU/mL and returned to normal after 6 weeks. However, the pelvic mass gradually increased. Re-examination of pelvic ultrasound performed showed a 7.7 cm × 7.4 cm × 8.2 cm mixed echoic mass on the right side of the uterus with clear boundary, fine punctate echo in the cyst, and abdominal and pelvic effusion. Contrast-enhanced ultrasonography showed that the cyst wall of the pelvic mass was significantly enhanced, malignancy was suspected, and surgical treatment was recommended. On November 14, 2018, the patient underwent transabdominal pelvic and intestinal adhesion release, omental, bilateral ovarian, peritoneal multipoint, and multiple adenomyomas, and hysteroplasty. During the operation, approximately 600 mL of hemorrhagic ascites were aspirated (Figs. 1 and 2). Upon exploration, a gray-red mass with a diameter of approximately 8 cm was observed on the surface of the greater omentum, which was soft and resembled rotten meat (Fig. 3). Diffuse “moss like” brown cellulose deposits were found on the surface of the uterus, bilateral adnexa, abdominal wall, intestinal tract, lateral groove of the rectum, and anterior wall of the rectum. The lesions were not completely cleared and some areas were electrocoagulated (Figs. 4 and 5). Pathological examination of the resected specimen showed retinal tissue endometriosis with vasodilation, blisters, scattered and focal lymphocytic infiltration, bilateral ovarian cystic follicles, peritoneal fibers inside adipose tissue necrosis, fibroblast proliferation, increased foam cell infiltration, and uterine adenomyoma (Figs. 6–8). Ascites pathology showed many red blood cells, a small number of small lymphocytes and neutrophils, and no tumor cells (Fig. 9). Postoperatively, the patient recovered smoothly, and was treated with gonadotropin-releasing hormone agonist (GnRH-α). Leuprorelin was started once a month for 6 months, followed by oral dienogest (DNG) for 3 years, after which the medication was stopped for 3 months. The patient conceived naturally and had normal pregnancy. A baby boy weighing 3300 g was delivered via vaginal discharge at 39 + 3 weeks of gestation. Both the mothers and babies were healthy, with no recurrence or adverse reactions during follow-up.

## 3. Discussion

A similar case was reported by Dun et al in 2016. A 26-year-old nulliparous woman of Nigerian heritage had recurrent hemorrhagic ascites due to endometriosis.^[10]^ Three years ago, the patient had undergone exploratory laparotomy for “pelvic mass and massive peritoneal effusion,” and 7 L of hemorrhagic ascites were evacuated from her abdomen. Endometriosis was confirmed by the biopsy of multiple lesions covering the peritoneum of the uterus, bladder, and pouch of Douglas. After the initial surgery, the patient was treated with goserelin for 3 months and oral medroxyprogesterone for 1 year, but failed to achieve pregnancy after the medication was stopped. The ascites reaccumulated after clomiphene ovulation induction therapy for 3 months, and leuprolide was administered again, followed by oral norethindrone. However, the condition did not improve significantly, and ascites persisted.^[10]^ A second laparoscopic exploration was performed and 7800 mL of hemorrhagic ascites was attracted. Diffuse olive-green “mossy” tissue covering the pelvic and abdominal peritoneum. Endometriosis was surgically resected using a combination of peritoneal stripping, excision with carbon dioxide laser, and ablation with neutral argon plasma. The intestinal adhesions were broken and the appendix was removed. The cytology of ascites revealed scattered hemosiderin-laden macrophages in the background of red blood cells, and densely distributed hemosiderin-laden macrophages were observed under a microscope in olive-green mossy lesions with few foci of endometriosis.^[10]^ Owing to the patient’s urgent fertility requirements, no medication was administered. The patient was still not pregnant until 6 months after surgery, and the ascites did not relapse.

The treatment can be considered similar to endometriosis: hysterectomy and bilateral oophorectomy are the only treatments with clear efficacy, and their indications are limited by the patient’s age and fertility needs. Surgical methods included ascites drainage, resection or fulguration of endometriotic lesions, adhesiolysis, hysterectomy, and unilateral or bilateral salpingo-oophorectomy. Owing to diffuse involvement of the peritoneal membrane and extensive abdominal adhesions, complete removal of the lesion is usually impossible, and GnRH-α treatment is required after surgery. However, ascites is prone to relapse after withdrawal.^[1,11,12]^ The control of pain symptoms in endometriosis requires the long-term use of progestins or oral contraceptives, however, the effect of medication on ascites remains uncertain. The management of infertility in such patients is more difficult, assisted reproduction is required,^[13]^ and ovarian stimulation may lead to recurrence of ascites.^[7]^

Due to extensive peritoneal lesions, surgery, hormones, or combined anti-inflammatory medications become the recommended treatment combination. Hormones include GnRH-α, GnRH antagonists, progestins, oral contraceptives, danazol and so on. DNG, a novel synthetic progesterone agonist, is a selective progesterone receptor agonist. DNG is as effective as GnRH agonist in relieving pain symptoms related to endometriosis. On the one hand, with significantly lower reduction of bone mineral density, and fewer hot flushes, which are the typical side-effects of GnRH agonists.^[11]^ In contrast, the long-term administration of low estrogen levels results in no obvious symptoms; however, there is a risk of breakthrough or irregular vaginal bleeding. Some researchers have shown that DNG could be an effective treatment for endometriosis-related ascites, which is an ideal choice for women who desire future fertility and may be used to replace long-term GnRH-α therapy for endometriosis. Other drugs, such as prednisolone, nonsteroidal anti-inflammatory drugs, pregabalin, and hormone combination therapy, may have a certain effect on controlling ascites.

## 4. Conclusion

In conclusion, ascites is a rare manifestation of endometriosis and is a group with severe symptoms. It is easily confused with other diseases that cause hemorrhagic ascites in clinical practice, and presents great challenges for clinicians in terms of diagnosis and treatment. Diagnosis is difficult, and laparoscopy or laparotomy is required to confirm it. Therefore, in clinical work, when the women of child-bearing age are complicated with a large number of recurrent ascites, after excluding other common diseases causing ascites, we should consider whether there is endometriosis combined with hemorrhagic ascites, especially pay attention to the presence of omental endometriosis.^[14]^ Periodic dysmenorrhea and abnormal menstruation should be suspected, and the location of the lesion should be carefully and comprehensively evaluated, particularly in the pelvic, umbilical, and right thoracic cavity. Patients with fertility needs and age adaptation should avoid hysterectomy and bilateral oophorectomy, as there is evidence that symptoms can also be successfully resolved by medical treatment.^[15,16]^ However, it is easy to relapse after medication withdrawal, and the optimal diagnosis and treatment strategy still needs to be further studied.

## Acknowledgments

The authors gratefully acknowledge the patients who agreed to participate in the study.

## Author contributions

**Conceptualization:** Feng Xu, Aihua Li.

**Data curation:** Feng Xu, Fengxi He.

**Formal analysis:** Feng Xu.

**Investigation:** Feng Xu, Yimeng Gao, Fengxi He, Miao Zhao.

**Supervision:** Yimeng Gao, Miao Zhao.

**Validation:** Yimeng Gao, Fengxi He.

**Writing – original draft:** Feng Xu.

**Writing – review & editing:** Aihua Li.
